# Trends on Aerogel-Based Biosensors for Medical Applications: An Overview

**DOI:** 10.3390/ijms25021309

**Published:** 2024-01-21

**Authors:** Cláudio M. R. Almeida, Beatriz Merillas, Ana Dora Rodrigues Pontinha

**Affiliations:** 1 University of Coimbra, CERES, Department of Chemical Engineering, Rua Silvio Lima, 3030-790 Coimbra, Portugal; claudio@eq.uc.pt (C.M.R.A.); beatriz.merillas@uva.es (B.M.); 2 LAQV-REQUIMTE, Departamento de Engenharia Química, Faculdade de Engenharia, Universidade do Porto, Rua Dr. Roberto Frias, 4200-465 Porto, Portugal; 3 Cellular Materials Laboratory (CellMat), Condensed Matter Physics Department, Faculty of Science, University of Valladolid, Campus Miguel Delibes, Paseo de Belén 7, 47011 Valladolid, Spain; 4 University of Coimbra, ISISE, ARISE, Department of Civil Engineering, 3030-788 Coimbra, Portugal; 5 SeaPower, Associação Para o Desenvolvimento da Economia do Mar, Rua Das Acácias, N° 40A, Parque Industrial Da Figueira Da Foz, 3090-380 Figueira Da Foz, Portugal

**Keywords:** aerogel, biosensors, biosensing, medical application

## Abstract

Aerogels are unique solid-state materials composed of interconnected 3D solid networks and a large number of air-filled pores. This structure leads to extended structural characteristics as well as physicochemical properties of the nanoscale building blocks to macroscale, and integrated typical features of aerogels, such as high porosity, large surface area, and low density, with specific properties of the various constituents. Due to their combination of excellent properties, aerogels attract much interest in various applications, ranging from medicine to construction. In recent decades, their potential was exploited in many aerogels’ materials, either organic, inorganic or hybrid. Considerable research efforts in recent years have been devoted to the development of aerogel-based biosensors and encouraging accomplishments have been achieved. In this work, recent (2018–2023) and ground-breaking advances in the preparation, classification, and physicochemical properties of aerogels and their sensing applications are presented. Different types of biosensors in which aerogels play a fundamental role are being explored and are collected in this manuscript. Moreover, the current challenges and some perspectives for the development of high-performance aerogel-based biosensors are summarized.

## 1. Introduction

Aerogels are a unique class of materials with a range of interesting properties, including low density (~0.001 g.cm^−3^), high porosity (ca. 80–90%), very low thermal conductivity, large surface area, high mechanical strength and they can reach translucency or even transparency, allowing light to pass through them [1,2,3,4]. These properties make them useful in a wide range of applications, including insulation, energy storage, catalysis, and biomedical engineering [5,6,7,8]. Aerogels are attractive materials for sensors due to their large surface area, porosity, and ability to be functionalized with biological molecules [9,10]. The highly specific surface area of aerogels and highly interconnected porous structure can ensure the adequate exposure of active sites, enhance the electron transfer, and supply adequate channels and abundant surface area for the transport and adsorption of analytes, thus endowing the resultant sensors with a fast response rate and high sensitivity [10].

A biosensor is a device that combines a biological component with a physicochemical detector to detect and measure the presence of specific biological or chemical substances [11,12]. The biological component of a biosensor can be a variety of elements, including enzymes, antibodies, nucleic acids (DNA/RNA) and whole cells [13,14,15]. These biological elements are chosen for their ability to selectively interact with the target analyte [16,17]. The physicochemical detector is responsible for converting the biological interaction into a measurable signal. According to the type of the transducer used, the biosensors can be divided into: electrochemical biosensors, optical biosensors, and mass-based biosensors [11,18]. The choice of the detection method depends on the nature of the biological component and the target analyte. The development and use of biosensors play a vital role in addressing multiple United Nations Sustainable Development Goals (SDGs) by contributing to health, environmental sustainability, and responsible technological innovation: biosensors are essential in healthcare for disease diagnosis, monitoring treatment efficacy, and managing chronic conditions (SDG 3); they are used in food safety and security to detect contaminants, ensuring the quality and safety of food products (SDG2); the production of biosensors can be made more sustainable by using environmentally friendly materials and manufacturing processes (SDG 12). These approaches have the potential to improve healthcare outcomes, reduce costs, and promote sustainability in the healthcare industry.

Aerogel-based biosensors have been developed for the detection of a range of biological molecules, including proteins, DNA, and viruses, relevant for diagnosis, disease monitoring, and other significant applications [9,19,20,21]. The aerogel matrix can be functionalized with specific biological molecules, such as antibodies or aptamers, which can bind to the target molecule. The binding event can then be detected using various transduction methods such as electrochemical techniques. Aerogel-based biosensors have several advantages over traditional biosensors, including increased sensitivity and selectivity due to their large surface area and porosity [21,22]. They also have potential for point-of-care diagnostics, as they can be designed to be portable and easy to use [23,24,25]. Aerogel-based biosensors enable early detection of diseases, leading to better health outcomes and improved well-being (SDG3). By offering real-time data, portability, and specificity, biosensors contribute significantly to advancing research, enhancing diagnostics, and improving safety and efficacy in various fields, such as medicine, biomedical applications, toxicology, ecotoxicology, food safety monitoring, drug delivery, and disease progression control.

**Figure 1:**
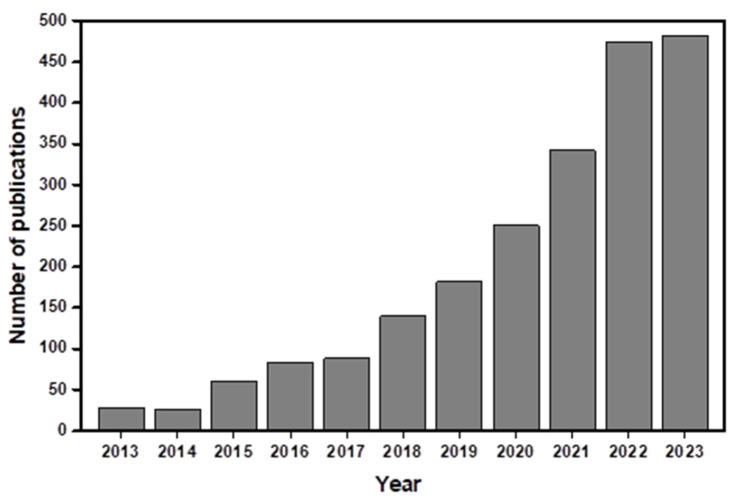
Number of publications (ScienceDirect record) during the last ten years containing ‘‘aero-gel” and “biosensors” in the content. (Date of search: November 2023).

As most of these works were published before 2018, and there has been a significant increase in publications since that year, as can be observed in Figure 1, the main goal of this review is to present the recent advances in the field, with a focus on the works developed with aerogel-containing materials for biosensing in the last 5 years.

In this review, the most representative information on aerogels used for biosensing applications is summarized. It is composed of four main parts: (i) a brief description of the main stages of aerogel preparation and their properties; (ii) a description of aerogels in sensors for biomedical application; (iii) an explanation of the recent advances of aerogel-based biosensors and (iv) main future trends and challenges for aerogels use in biosensing.

## 2. Chemistry of Aerogels and Their Properties

Aerogel is a material discovered in the 1930s [26] and well-known by its three-dimensional nanoporous structure [27,28,29]. Based on their chemical compositions, aerogels can be classified as organic (cellulose, pectin, resorcinol formaldehyde, polyurethane), inorganic (silica, titania, alumina), carbon (via pyrolysis of organic aerogels), and hybrid/composite aerogels. Aerogels have low density, high porosity, large surface area, and open porous structure [30]. These unique properties make them very attractive for several applications such as tissue engineering [31], drug delivery [32], sensors [10], adsorption [33], catalysts and supports [34,35], thermal insulation [36], acoustic insulation [37], solar systems [38], and energy conversion, and storage applications [39]. Regarding sensor applications, the lightweight and highly interconnected porous structure and large specific surface area of aerogels can prevent aggregation of the sensing materials, which ensure an adequate exposure of the active sites for the target analytes. Additionally, the existence of channels and abundant surface area lead to high transport and adsorption capacity for the analytes, thus ensuring a fast response rate and high sensitivity.

The properties of aerogels can be tuned by adjusting the synthesis conditions, precursor materials used, and drying method, making them a versatile material for a wide range of applications.

### 2.1. Aerogels Production Process

**Figure 2:**
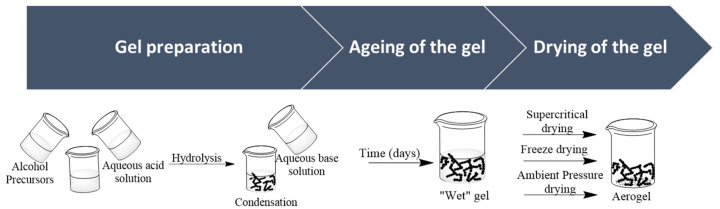
Schematic representation of a typical silica sol-gel synthesis procedure, with two-step acid-base catalyzed process.

Aerogels are amorphous materials composed of an open and porous three-dimensional silica network. These materials are usually prepared by the well-known sol-gel process [3,28,40], which promotes, at low temperatures, the synthesis of their solid network by chemical reactions in solution [29,41]. The synthesis of aerogels can be summarized in three main steps: (i) gel preparation; (ii) aging; and (iii) drying, as illustrated in Figure 2.

As the first step, the wet gel is formed as a result of hydrolysis and condensation reactions of the silica precursors, usually silicon alkoxides such as tetramethoxysilane (TMOS) and tetraethoxysilane (TEOS) for silica aerogels [3]. In the hydrolysis reactions, the alkoxides are converted into silanols, and, once hydrolyzed species are present in the solution, condensation reactions begin to happen. These reactions form siloxane bridges (Si-O-Si) [42,43], leading to the development of a nanostructured solid silica network. These reactions are usually catalyzed by acid and/or basic catalysts, as the solution pH has a major impact on the gels’ microstructure [43]. If they are acid catalyzed, hydrolysis is faster than condensation, and a less branched silica network is developed, while if basic catalysts are used, the condensations reactions are favored, leading to the formation of highly condensed and ramified networks [28,44,45]. When the gel point is reached, i.e., the system loses fluidity and viscosity increases sharply, a solid object is obtained, and is then assumed that hydrolysis and condensation are nearly complete [28,42,46]. Graphitic materials, such as carbon nanotubes and graphene, can also be precursors to form gels (crosslinked by Van der Waals interactions, π-π interactions and/or H-bonds) for carbon aerogels, while the synthesis involves using metal alkoxide precursors, which undergo hydrolysis and condensation reactions to form the metal oxide gels [47].

The second step of aerogels’ synthesis is aging. In this process, hydrolysis and condensation reactions are still happening, although at a much lower rate, leading to a strengthening and stiffening of the network [48,49]. Two main mechanisms simultaneously occur during this stage: neck growth from reprecipitation of dissolved silica, causing the thickening of the interparticle bridges; and the Ostwald ripening phenomenon, in which the dissolution/re-precipitation of silica particles takes place around bigger particles [3,28,48]. These mechanisms cause the gel structure to be more rigid and cohesive with the increase in aging time and proper solvent choice [3,46].

The final step is gel drying, whose main objective is to remove the solvent while preserving the gel’s pore structure. For many years, supercritical drying was the most used approach to obtain aerogels, since eliminates capillary forces, thus avoiding significant shrinkage/collapse and achieving minimal impact on the structure of the gels network [3,27,28]. However, due to the high costs and safety concerns of this technique [29], different strategies have been developed in order to obtain samples with the same features as the ones obtained by supercritical drying methods, but with simpler and easier to scale-up procedures [27].

One way of accomplishing this goal is to perform a surface chemistry modification in the material prior the drying step. The surface modification can be achieved by two main routes: co-precursor method, in which a precursor having non-hydrolyzable organic groups, such as trimethylethoxysilane (TMES) and methyltrimethoxysilane (MTMS) [50], is added into the precursors solutions before gelation; and the derivatization method, in which the surface modification is performed after gelation, by immersing the wet gels in an organosilane solution, containing trimethylchlorosilane (TMCS) [51] for example, which causes the organic groups to attach to the wet gel skeletons [52]. These modifications, besides preventing further condensation from taking place due to the suppression of reactive groups in the alcogel structure, also decrease the surface tension [3,29]. Thus, they allow the material to have properties similar to the ones observed in their supercritically dried counterparts, while using evaporative drying in ambient pressure conditions.

### 2.2. Aerogels Surface Properties

Aerogels possess unique properties that make them highly interesting and versatile materials. They can present hydrophobic or hydrophilic surface, depending on the synthesis process and surface modifications. The major drawback regarding native silica aerogels, is the mechanical strength and their intrinsic fragility, which significantly limits their practical applications. Therefore, different techniques and materials, from polymers to nanostructures, have been reported in the literature to mechanically reinforce silica aerogels [2,3,53,54].

#### 2.2.1. Silica-Based Aerogels

The sensing elements for target recognition devices need to be incorporated in a matrix to form a sensor. Due to their exceptional properties, silica and carbon matrices can be suitable for this purpose. The silica matrix is typically obtained through the sol-gel process, a type of bottom-up synthesis approach for nanostructured networks, performed in a liquid medium at temperature typically bellow 100 °C [42].

**Figure 3:**
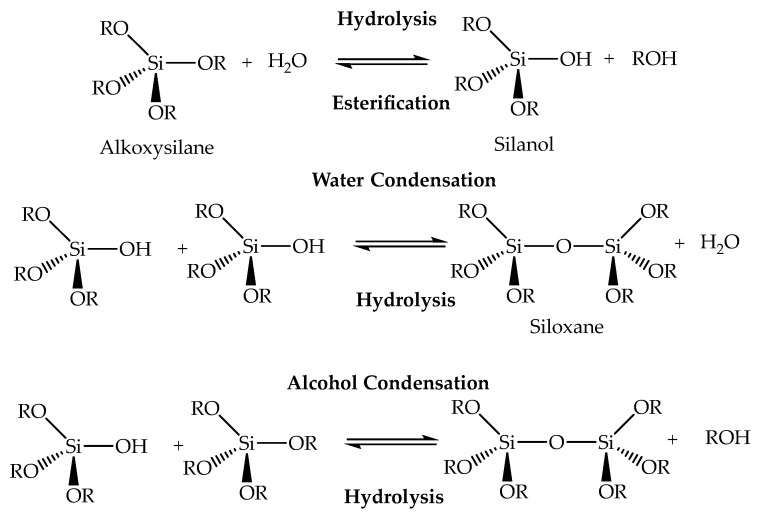
Steps of the sol-gel chemistry in silica systems.

In the case of silica gels, they result from the polymerization process involving the formation of Si–O–Si bridges (siloxanes) through the hydrolysis and condensation/polycondensation reactions of the silica precursors, Figure 3. The change from a liquid to a solid phase is called the “sol-gel transition”.

The use of acid or alkaline conditions, the amount of water for hydrolysis reactions, the temperature, as well as the nature of organic groups of the precursor have a large influence on the sol-gel process [55,56]. By controlling these conditions, materials with different properties can be obtained according to the intended applications.

The sol-gel methodology opens the possibility to incorporate organic moieties into inorganic materials, creating hybrid materials [57,58]. This strategy can be performed by two different strategies: (i) using organo-substituted silica precursors during the synthesis procedure (co-precursor approach) or/and (ii) by surface modification after the gel formation (silylation or surface derivatization) [57,58,59]. In the silica systems, the presence of a non-hydrolyzable organic group will confer specific properties to the materials. Tetraethylorthosilicate-based materials exhibit high hydrophilicity due to the hydroxyl groups at the network ends. Replacing these groups with organic ones using an organosilane such as methyltrimethoxysilane, for example, will originate –CH3 groups on the material’s surface, that will give a hydrophobic character to the final material.

The aminosilanes, which are characterized by having one or more amine groups, are a class of organosilanes with important properties [60]. This group is responsible for the high reactivity behavior of these precursors. The electronegative nitrogen atom of the amine group can enter into hydrogen bonding interactions with hydrogen donating groups, such as hydroxyl groups or other amines [60]. The amino groups also play an important role as active sites on the silica matrix to bind other molecules or species, creating new interactions and functional points on the surface. This capacity can be explored in fields like environmental remediation, catalysis and sensors [61,62,63]. For these reasons, there is great interest in the development of new applications of amino-modified silica matrices.

In sensor design, other important organically modified silanes are the carboxyl substituted silanes. Carboxyl groups (–COOH) can be used to link specific elements, such as antibodies, DNA sequences or other molecules [64]. Feinie and co-workers reported the use of 5-(triethoxysilyl)-pentanoic acid as a precursor molecule and Cetyltrimethylammonium bromide (CTAB) to obtain carboxylic acid group-containing silica materials, using a co-condensation approach [65]. The high versatility of the silica sol-gel process allows a large number of options and strategies regarding the material functionalization with different groups, enabling a wide range of applications, including sensors and biosensors [10,57].

#### 2.2.2. Carbon-Based Aerogels

**Figure 4:**
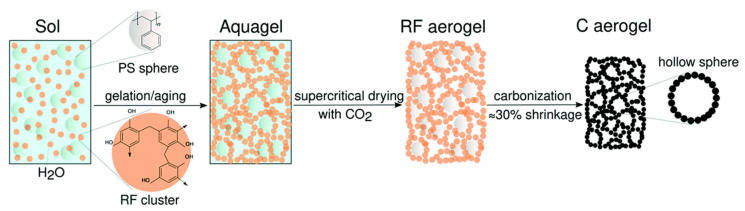
Illustration of carbon-based aerogel synthesis process [67].

Carbon aerogels are a type of nanostructured material that has received increasing attention since the 1990s. They were first synthesized by Pekala using resorcinol and formaldehyde as precursors [66], Figure 4.

Nowadays, carbon aerogels can be obtained with carbon nanomaterials, resulting in materials with high porosities (90–99%), large surface area (500–2500 m^2^/g), ultralow density and remarkable chemical, thermal and mechanical stabilities [9,67]. Due to the above-mentioned properties, along with conductivity and biocompatibility, carbon aerogels are excellent candidates for electrochemical biosensors development. They can provide multiple accessible sites to be functionalized with sensing elements, and favoring the adsorption and transport of target analytes, thus providing the sensing probes with high sensitivity and fast response rate [9].

Carbon-based nanomaterials have been the subject of intense research due to their unique structural and physical properties and manly because their intrinsic electronic, magnetic and optical properties [68,69]. The chemical versatility of these compounds is another important aspect regarding sensors development. Carbon nanomaterials, composed of sp2 bonded graphitic carbon, are found in different dimensionalities, including zero-dimensional (carbon dots) and one-dimensional (carbon nanotubes) [69].

The intrinsic properties of carbon nanomaterials allow the employment of different sensing mechanisms such as optical and electrochemical, due to fluorescence quenching resulting from the interaction with the analyte, and redox process at the carbon material’s surface [69]. However, there are some aspects to be considered, regarding the incorporation of these materials in water containing formulations. In general, these materials display poor dispersion in aqueous solutions, where they tend to agglomerate due to hydrophobic interactions. One of the simple methods to overcome this problem is through surface functionalization by covalent or non-covalent methods [70]. The simpler ones rely in the dispersion of these structures in the aqueous phase by sonication and use amphiphilic molecules or surfactants. In the case of sp^2^ carbon species, covalent functionalization results in the permanent disruption of the π-electronic network, which can influence the optical properties of the material [70]. This can be avoided through the use of molecules that modulate π-π interactions such as porphyrins or other optically active molecules. However, covalent functionalization is still the most effective method for the stable dispersion of carbon structures in aqueous solutions.

Surface modification can also be used to introduce active sites for sensing purposes. For example, carboxyl groups produced by oxidation in an acid medium do not significantly affect the optical and electronic properties. These carboxyl groups can later react with alcohols, phenols, alkyl halides, anhydrides, or amines. Other reactive functional groups, such as amino, hydroxyl, and alkyl halogens can be introduced on carbon nanostructures by chemical reactions [70]. Further, the reactive functional groups introduced can be used for the conjugation with a wide range of elements such as organic dyes, DNA sequences, proteins, and nanoparticles, among others. The covalent bonding is possible due to the COOH groups present on the electrode surface, which were converted into the reactive succinimidyl esters using a 1-ethyl-3-(dimethyl-aminopropyl) carbodiimide hydrochloride (EDC)/N-hydroxyl succinimide (NHS) solution [71].

## 3. Aerogels-Based Sensors for Biomedical Applications

Aerogels offer excellent research opportunities in the medical and pharmaceutical fields for drug delivery, as antifungal or antibacterial materials, tissue and bond regeneration, or even for biosensing and bioimaging [72]. The high sensitivity and selectivity of aerogels when applied as biosensors for detecting biological molecules expand their applicability to these sectors, where detecting remarkably low concentrations of certain biomolecules is crucial for diagnosis. Aerogels can be used as substrates, electrodes, or matrices for biosensors, taking advantage of their unique properties, such as their large surface area, huge porosity, tenable pore size, and good biocompatibility [7,8].

Some aerogel-based sensors have been used on the biomedical field for detecting toxic gases for disease diagnosis. The structural interconnection that aerogels present, with an open porosity, provides a fast path for diffusion of harmful gas molecules such as NO_2_, SO_2_, or H_2_S [73,74,75]. Other promising sensors in this field are piezoresistive sensors based on aerogels. The capacity of being deformed without breaking and the certain elasticity that some matrixes provide, allow the use of these sensors covering a wide sensing range. These sensors have been currently used for monitoring human motion (voice, body, face, etc.) for health detection [76,77]. Nevertheless, the current trend is moving to biosensors that detect target molecules with a high signal amplification, accuracy, and rapid diagnosis [78,79]. The main contributions to the biomedical sector employing aerogels for biosensing have been gathered in the following paragraphs and classified according to the aerogel matrix type.

The following table, Table 1, gathers some recent work on biosensors for acquiring different signals for medical diagnosis.

### 3.1. Carbon-Based Aerogels

Carbon-based aerogels are explored in biomedical engineering owing to their high potential and formation of joints with biocompatible metals [9,98]. In 2019, Chen et al. [80] developed carbon aerogel wearable sensors from bacterial cellulose that monitored biosignals of the human body. These materials presented a high linear sensitivity in a strain range from 0 to 95% owing to their characteristic structure formed by oriented wave-shaped carbon layers. More recently, in 2021, Yang et al. [81] proposed a nature-derived pH sensor for monitoring chronic wounds based on 3D conductive carbon nanofiber aerogel as a substrate obtained from bacterial cellulose pyrolysis. For inducing proton-selectivity, polydimethylsiloxane/polyaniline (PDMS/PANI) composite was included into the substrate reaching a pH sensitivity of −50.4 Mw/pH in the buffer solution and −29 mV/pH in in vitro simulated wound fluid. Sensitivity is affected by serum protein through proton- binding; however, the obtained results suggest that this sensor could be applied as a smart wound dressing after further optimization.

Other carbon forms are found in biosensors, such as graphene oxide aerogels [82] for detecting a flavonoid drug; quercetin in tablets, graphene aerogel for detecting glucose in the prevention and diagnosis of diabetes [86], or multi-walled carbon nanotube hybrid nanostructured aerogel-like material for electrochemical dopamine detection in biological samples (rat brain, human blood serum, dopamine hydrochloride injection) [83]. Liu et al. [84] synthesized sandwich-type 3D graphene aerogels loaded with gold nanoparticles for their use as substrates of an electrochemical immunosensor for quantitative detection of carcino embryonic antigen (CEA). The produced materials showed a large specific surface area (1332 m^2^/g), good biocompatibility and stability, properties that allowed the effective immobilization of antibodies. Then, to reach an enhanced sensitivity, quaternary chalcogenide nanocrystals—Cu_2_ZnSnS_4_ were used as labels, reaching a lower detection limit (0.15 pg/mL (S/N = 3)) in combination with high sensitivity and selectivity. These biosensors showed an effective electron transfer electrode-electrolyte, better than the convectional 1D or 2D carbon electrodes that present lower specific surfaces. A similar sensor produced with graphene oxide/Au aerogels was used for detection of uric acid in human sweat obtaining results comparable to the performance of liquid chromatography, with a very low limit of detection (3.7 μM) [85].

It is well-known that early diagnosis and treatment of some diseases is of utmost importance. For instance, Ruiyi et al. [87] developed an electrochemical sensor based on graphene aerogel microspheres able to detect liver cancer cells in blood. The high efficiency of this sensor (low detection limit of 5 cells/mL) presents a great potential for cancer diagnosis.

### 3.2. Silica-Based Aerogels

Silica aerogels have a special place in nanotechnology because of their structural tailorability, precursors diversity, huge surface area, and being used in high-tech science and engineering [99]. In 2001, bacteria were immobilized on silica aerogels for biosensors that detected viral particles [100]. Some years later, amino-silica aerogels were prepared for the development of biochips for antigens detection (human IL6) achieving significant improvements in the detection limit in comparison with planar 2D biochips (from 0.06173 ng/mL for planar to 0.06173 ng/mL for the aerogel biochip) [88]. The same authors synthesized a 3D silica-aerogel biochip for recognition of nucleotide acids obtaining a larger capturing capacity than other sensors with planar surface [89].

### 3.3. Polimeric Aerogels

Cellulose nanomaterials have been widely used in biological studies and biomedical applications [101,102]. The effective surface area of cellulose nanofiber-aerogels expands their use to biosensing research as described in the following works. In 2016, Edwards et al. [90] designed peptide-nanocellulosic aerogels from unprocessed cotton treated with a fluorescent tripeptide-substrate for protease detection. The peptide was immobilized on the surface and the detection sensitivity of neutrophil elastase was 0.13 units/milliliter, thus it became a biomarker of inflammatory diseases and showed a 4-fold affinity improvement than the free peptide substrate. Then, other biosensors based on nanocellulose were reported for detecting heavy metals in human serum [91].

Zhang et al. [92] developed a portable and non-invasive point-of-care (POC) sensor for alcohol detection. The poly(vinyl alcohol) aerogel matrix was loaded with Pd@Pt nanoparticles and alcohol oxidase (AOX) for catalyzing the transformation from alcohol to oxygen through a peroxidase-like activity. Saliva and blood can be quicky absorbed by the composite and the alcohol quantity can be measured by a gas pressure meter, reaching a limit of detection lower than the legal limit for driving (DL of 0.50 mM in saliva,). Moreover, the AOX can be replaced by glucose oxidase leading to an accurate detection of glucose, thus expanding the use of this sensor.

### 3.4. Metal-Based Aerogels

Currently, some metal-based aerogels have been employed in biomedical sensing with the aim of enhancing the selectivity, stability, and sensitivity of the already developed planar electrodes owing to their unique physiochemical properties. Noble-metal aerogels, especially those of Au, Ag, Pd, and Pt, show significantly better activity towards the detection of different bioanalytes than their counterparts. Nevertheless, there are some concerns regarding their biocompatibility, since in general these metals are nontoxic for cells and microorganisms, their ions are usually toxic. Thus, research is exploring the use of these aerogels in biosensing and bioimaging applications while overcoming the current challenges [103].

For instance, Guan et al. [93] produced a gold nanowire aerogel that detected ethanol in a sweat matrix with high accuracy, quick linear response, and an excellent performance. Wen et al. [94] immobilized glucose oxidase on palladium aerogels with the aim of oxidizing glucose for potential biosensors. These materials reached a superior sensitivity than other sensors, as well as 12 h stability under continuous operation.

Wu et al. [95] combined two noble metals by synthesizing polydopamine-capped bimetallic AuPt Hydrogels. This matrix provided good biocompatibility, high porosity, and large surface area being superb platforms to enzyme immobilization. By immobilizing acetylcholinesterase (AChE), toxic and harmful organophosphorus compounds were detected.

### 3.5. Hybrid Aerogels

Combinations of different aerogel matrixes have been applied for synergistic effects between them. For instance, Sun et al. [96] combined nanosilica and graphene oxide for producing a selective and sensitive sensor for detecting insulin. Owing to the low detection limit of this biosensor, it was successfully used in insulin injection samples with a high recovery (98–103%). More recently, in 2023, a nanocomposite aerogel combining graphene oxide and polyurethane was fabricated by Zhang et al. [104] for monitoring the response of electronic skins to different physical signals. The unique characteristics of this sensor are their ultrabroad-range response (1 Pa–12.6 MPa), superelasticity (90–99% reversible strain), and reusability (withstanding 10 000 compression cycles under 1 MPa).

CNTs/MoSx aerogels produced via solvothermal method were able to immobilize H7-polyclonal antibodies showing an effective detection of the Avian Influenza Virus H7 with a very low detection limit of 0.43 ng/mL [97].

**Figure 5:**
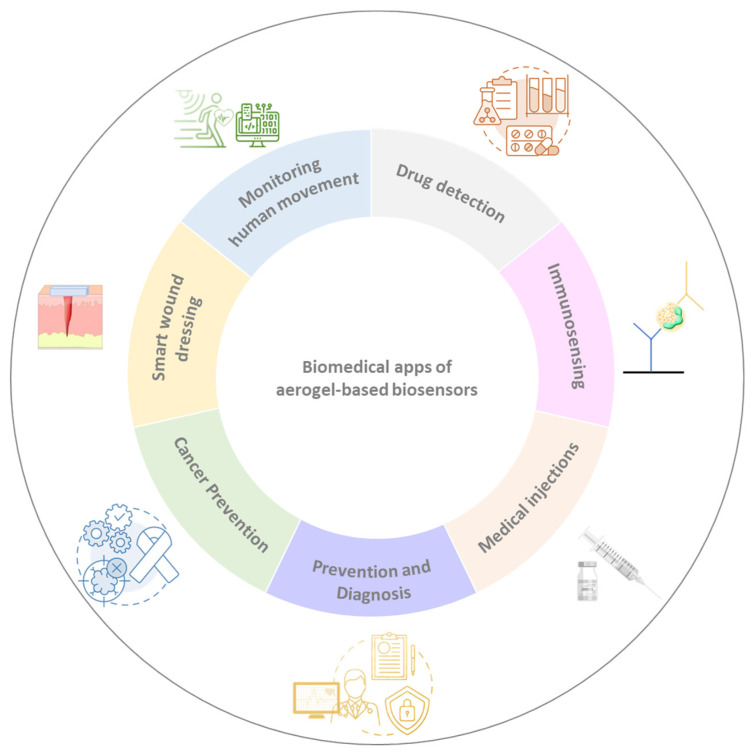
Main applications in the biomedical sector of aerogel-based biosensors.

Thus, aerogel-based materials produced with different matrixes provide a successful substrate for biosensors presenting biocompatibility with the complexity of the bio-system they are applied to. The incorporation of biomaterials in these aerogels has demonstrated to be an excellent strategy providing mechanical and chemical robustness for the complex biological matrixes usually found in the biomedical field (wound fluids, blood matrixes, tissues, etc.) thus expanding their application, shown in Figure 5. Therefore, aerogel-based biosensors are promising materials for biomedical applications, such as diagnosis, virus and bacteria detection, as therapeutic tools, or even for medical treatments.

## 4. Recent Advances in Aerogel-Based Biosensors for Sensing Applications

A biosensor is an analytical device that combines a biological component with a physicochemical detector consist of a sensing bioreceptor, a transducer; and a detector with a digital output [105]. The transducer recognizes the analyte through the reaction and transforms the signal to translate molecular changes into a quantifiable signal. The recognition elements in a biosensor are immobilized onto the surface of transducers, allowing them to interact with target molecules without adding reagents into the sample solution. In operation, the specific interactions between the target analyte and the recognition elements would produce physicochemical changes on the transducer surface. The changes are then recognized by the transducer, and converted into measurable signals which then could be used to determine the amount of analyte that is present in the sample. Generally, biosensors are classified based on either the biological component used, such as enzymes, antibodies, or nucleic acids, or by the type of transducer, such as electrochemical, thermometric, optical, mass-based, (acoustic or piezoelectric) and magnetic transducer. In this work we will use the first classification.

### 4.1. Enzymes-Based Biosensor

Enzymes were the first specific molecular component to be used as biosensors, often embedded within surface structures, allowing for short diffusion pathways between biorecognition element and transducer. Enzymes constitute an extremely important class of biomacromolecules with diverse catalytic functions, which have been validated as key mediators for regulating cellular metabolism and maintaining homeostasis in living organisms [106]. Their activity goes from cell energy, drugs, and toxicants metabolism, cellular homeostasis, to the development of different diseases. Enzyme-analyte specificity is achieved by using the binding cavities within their 3D structure and non-covalent interactions, such as hydrogen-bonding and electrostatic, to form recognition patterns with the target. Enzymatic biosensors are biocatalytic in nature since the enzyme captures and catalytically converts the target analyte into a measurable product.

**Figure 6:**
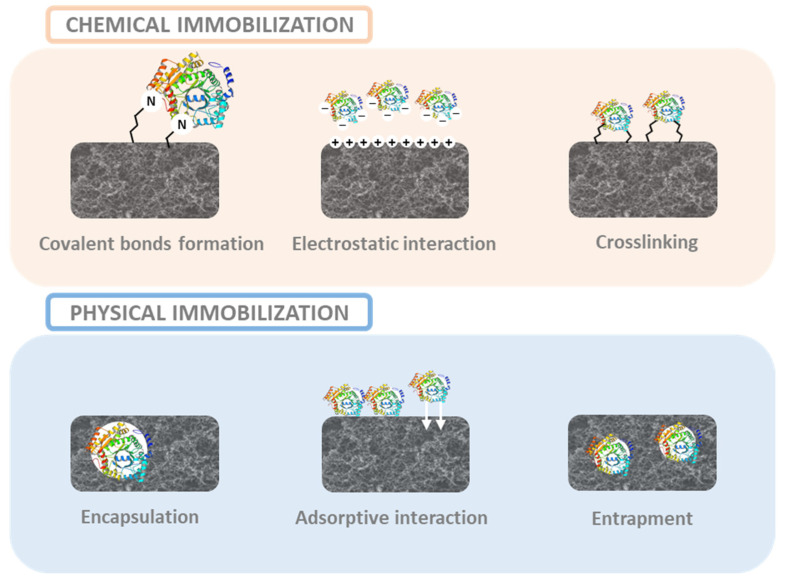
Different enzyme–immobilization techniques.

Enzymes not only have been used for triggering biochemical cascade reactions, environmental monitoring, and food quality control, but also as biomarkers for human diseases which implies a huge potential for medical diagnosis [107,108,109]. Thus, these molecules are being used for the development of enzyme-based biosensors for detecting target analytes in complex systems [110]. One of the main challenges is achieving an effective enzyme immobilization into a matrix with a large surface area while providing a good mechanical stability. Therefore, different immobilization techniques have been developed consisting on entrapment, adsorption, and encapsulation mechanisms (based on physical interactions), or crosslinking, covalent bonds formation, and electrostatic attraction (based on chemical interactions) [111], as displayed in Figure 6. Regarding the possible supporting materials, nanomaterials are placed on the top owing to their huge specific surface area, thus, aerogels being interesting and promising candidates. The following table, Table 2, summarizes the main works of enzyme-based aerogels published in the recent years.

The activity of certain enzymes has been mimetized by using different aerogel matrixes. For instance, an enzyme-free photoelectrochemical sensor was produced by Li et al. [112] simulating peroxidase activity by employing a AgCu@CuO-based aerogel. It allowed the evaluation of the xanthine oxidase activity with a significantly low detection limit of 65.23 nU/mL. Other enzyme-free aerogel sensors have been investigated by including nanoparticles [113] and chemical compounds [114] for glucose and hydrogen peroxide detection.

**Table 2:** Enzyme-based biosensors in aerogel matrices..

Aerogel Matrix	System	Target	Detection Technique	Lineal Range	LOD	Reference
Silver nanoparticle/graphene	Sulfite oxidase enzyme (SOx) immobilized on silver NP	Sulfite	Electrochemical	0.025–40 mg/L	0.007 mg/L	[21]
Graphene/Au NP	Cytochrome c (Cyt c) immobilized in 3D graphene aerogel with Au nanoparticles (AuNPs)	H_2_O_2_	Electrochemical	10–740 μM	1.1 μM	[115]
Graphene	Glucose oxidase immobilized on a graphene aerogel	Glucose	Electrochemical	1–18 mM	0.87 mM	[86]
UiO-66-NH2	Glucose oxidase immobilized on UiO-66-NH2 aerogel containing an iron porphyrin	Glucose	Colorimetric	10–400 μM	0.3 μM	[116]
Poly(vinyl alcohol) (PVA)	multienzyme immobilization	Glucose	Colorimetric	-	11.4 μM	[117]
Au	Au hydrogel nanozyme with glucose oxidase and peroxidase-like activity	Glucose	Colorimetric	5–80 mM	1.65 mM	[118]

NP = nanoparticles; LOD = limit of detection (S/N = 3).

Nevertheless, some enzyme-based aerogels were used for sensing applications in the recent years, as can be found in Table 2.

**Figure 7:**
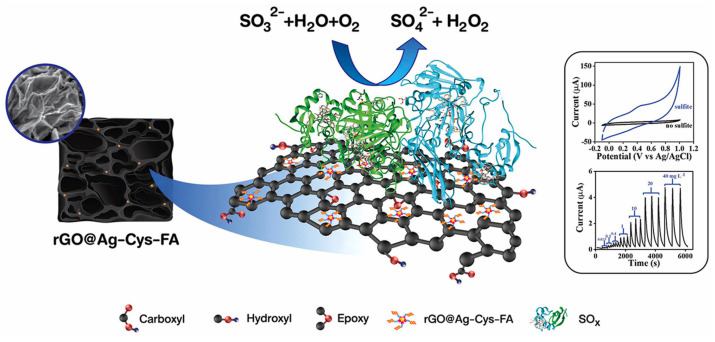
Amperometric sulfite detection by an enzyme-based 3D silver nanoparticle/graphene aerogel biosensor [21].

Electrochemical sensors employing a graphene aerogel matrix with immobilized enzymes have been fabricated. In 2022, Sroysee et al. [21] successfully immobilized sulfite oxidase on a graphene aerogel containing silver nanoparticles, as can be seen in Figure 7. These nanoparticles had a twofold commission; serving as anchoring points for the enzyme while providing a high electrical conductivity, allowing to produce an electrode for continuous amperometric sulfite detection. The immobilization method was the formation of amino-linkages between the enzyme and substrate.

Additionally, Zhao et al. [115] used biocompatible golden nanoparticles to immobilize Cyt C in the large-surface graphene aerogel. This enzyme promoted the electron transfer on the electrode, providing a highly sensitive sensor for hydrogen peroxide detection. Xu et al. [86] also produced an electrochemical biosensor by immobilizing glucose oxidase on a graphene aerogel produced by freeze drying. The high electrical conductivity reached by the material led to a low limit of detection (0.87 mM) reaching a high selectivity and good recovery. Therefore, constituting promising materials for clinical diagnosis of different diseases in the medical field.

**Figure 8:**
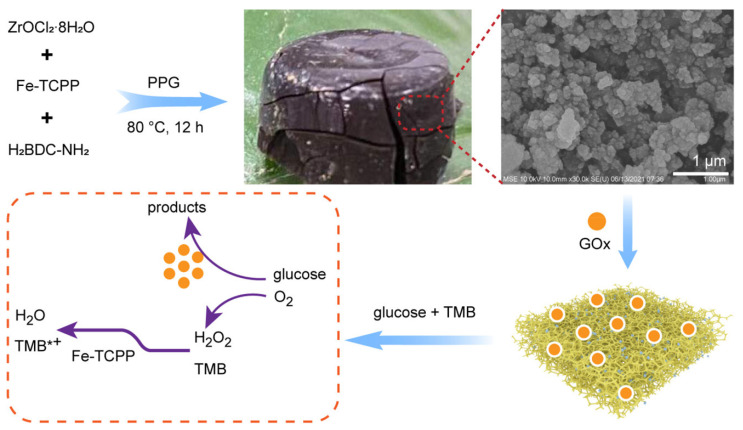
MOA-immobilized enzyme for glucose detection [116].

Apart from electrochemical sensors, colorimetric biosensors have been synthesized with different aerogels for glucose detection. Metal-organic aerogels (MOAs) were employed for the production of enzyme biosensors, Figure 8. A zirconium MOA (UiO-66-NH_2_ aerogel) was doped with an iron porphyrin to promote the attachment of glucose oxidase [116]. The system demonstrated effective detection of glucose using colorimetric sensing, with a low detection limit and great selectivity. Additionally, this sensor provided an improved stability in organic solutions thanks to MOA, which acts as a support to protect the enzyme.

**Figure 9:**
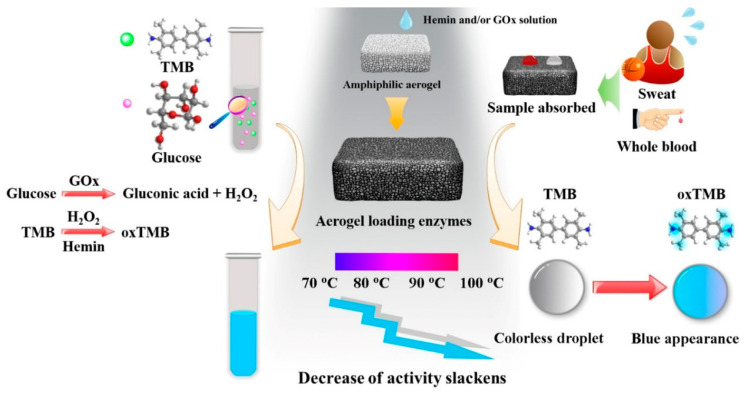
Scheme of enzyme immobilization and final properties of PVA aerogel biosensor [117].

Other colorimetric sensors for glucose determination can be found in the literature based on gold and poly(vinyl alcohol) (PVA) matrixes. Ma et al. [117] used a polymeric matrix of poly(vinyl alcohol) in combination with maleic acid for modifying the aerogel surface as substrate for a multienzyme complex, Figure 9. This matrix stabilizes the enzymes, avoiding their degradation at high temperatures, while preserving their conformation. Glucose oxidase and hemin were immobilized for catalyzing cascade reactions. The developed composite was able to detect glucose in blood and sweat samples as well as providing a significant structural stabilization.

Despite the relevant activity of enzymes, several intrinsic drawbacks exist in the employment of these biomolecules, such as high-temperature deactivation, high cost, or poor recyclability. Thus, in the last decade, there has been a trend based on synthesizing “artificial enzyme-mimicking nanomaterials”, also called nanozymes. Nanozymes present several advantages in terms of cost-effectiveness, durability, and chemical stability, widening the range of applications. They can be classified into nanomaterial hybrid enzymes in which enzymes are assisted by a material that improves their stability, and nanomaterials with an inherent enzymatic activity that play a similar role than enzymes [119]. Following this tendency, Jiao et al. [118] proposed an efficient method for fabricating a gold hydrogel constituted by a nanowire structure. Polydopamine induced the self-assembly of the hydrogel, leading to a material with an efficient electron transfer that shows glucose oxidase-like and peroxidase-like activity.

### 4.2. Antibodies-Based Biosensor

Antibodies are naturally occurring 3D protein structures, typically ~150 kDa in size, that have been widely used as a capture probes in biosensors, as they have naturally evolved to bind their target analyte with high affinity and specificity.

**Figure 10:**
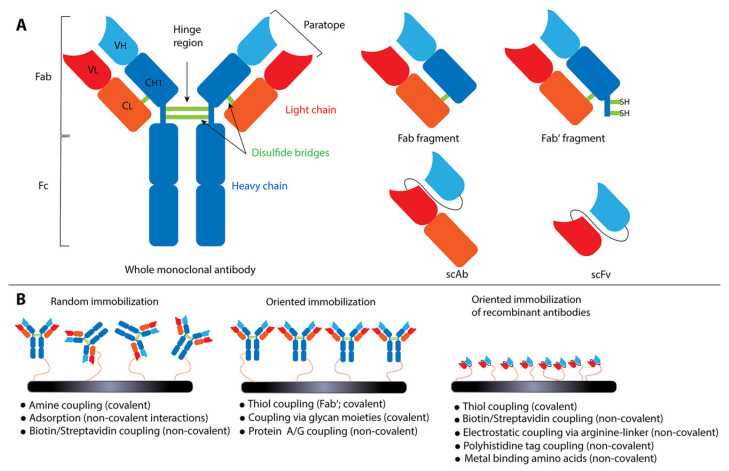
Antibodies as capture probes in immunosensors. (**A**) Schematic overview detailing the structure of whole antibodies and their fragments such as Fab, Fab’, scAb and scFv. (**B**) Strategies for immobilization of antibodies and their derivatives onto biosensor surfaces [128].

Antibodies are proteins crucial in the adaptive immune system of vertebrates targeting antigens such as pathogenic microorganisms, and they are one of the most important class of biorecognition elements [120,121]. They are usually immobilized via covalent linkage to a sensor surface, forming a brush-like array. Antibodies have a general structural trend of a “Y” shaped, which creates a unique recognition pattern with high specificity and selectivity for the analyte. They are affinity-based bio-recognition elements so the obtained signal is dependent on the binding event to form an antibody–antigen immunocomplex. Antibodies consist of two moieties bound together by disulphide bonds—the hinge region [122,123], Figure 10A. Each moiety contains a light and a heavy chain. The light chain has one variable region (variable light or VL) and one constant region (CL) and the heavy chain is composed of one variable region (variable heavy or VH) and three constant regions (CH1, CH2 and CH3) [122]. Antibodies can be divided into five classes depending on their heavy chain constant region sequences, i.e., IgM, IgD, IgG, IgE and IgA [124].

Antibodies and their derivatives, such as antigen-binding fragments (Fab, Fab’), single-chain variable fragments (scFv), or single-chain antibodies (scAb), are extensively employed as capture probes in biosensing structures, and the resulting biosensors are appropriately referred to as immunosensors [125,126].

The antibody immobilization onto the sensor surface is key for maintaining its proper conformation and correct orientation to permit best interaction with the target analyte [127], Figure 10B.

There are different routes to immobilize the antibody on the surface of the sensor: side-on (one Fab’ and one Fab attached to the surface), tail-on (Fab’ attached to the surface), head-on (both Fabs attached to the surface) or flat-on (all three fragments attached to the surface). Adsorption and amine coupling or covalent and oriented immobilization of antibodies may be necessary [128].

As a valuable alternative, biosensor incorporating aerogels can be regarded as a feasible strategy for the rapid, specific and sensitive detection of antibodies at the point-of-care. Table 3 summarizes the antibody-based biosensors, incorporating aerogels in the system.

A sensitive sandwich-type biosensor using an electrochemical immunoassay system for the multiplex sensing of AFP and CEA was studied by Filik et al. [129]. They used AuNPs that were anchored to ethylenediamine-MWCNT aerogels (EDA-CAGs) employed as the biosensing platform. The synthesized AuNP-Thi-EDA-CAG and AuNP-SfO-EDA-CAG complexes, owing to the redox-active species (Thionine and Safranine O) were successfully employed as electrochemical immunosensor. The prepared AuNP-EDA-CAGs provided a huge active surface region for the assembling of the capture antibody and aided the electron transfer on the SPCE surface. Furthermore, the synthesized AuNP-Thi-EDA-CAG and AuNP-SfO-EDA-CAG architectures possessed appropriate bioactivity to accelerate the electron transfer and improved the immobilization amount of detection antibodies. The synthesized EDA-CAGs enhanced the immobilization of detection and capture antibodies. The results demonstrated that the composed sandwich-type immunoassay protocol performed suitable reproducibility, high sensitivity, good precision, and resistance.

Tang et al. improves the sensitivity of lateral flow immunoassays devices with the aim to lower the limit of detection and to facilitate quantitative analysis [130]. They showed that by integrating a CNF aerogel intermediate stack layer into a conventional LFIA device, the sample flow time is effectively increased and, as a consequence, an extended interaction time between the bioreagents results in up to a 1000-fold improvement of the limit of detection for mouse IgG.

Jia et al. fabricated 3D Au–PEDOT–graphene aerogels using the electrodeposition of Au on the surface and the in-situ polymerization on 3D graphene aerogels [131]. This composite nanostructure is a highly sought-after material with a 3D PEDOT-based surface via PEDOT coating of the 3D graphene skeleton. Antibodies for detecting prostate cancer factors were introduced on top of this material to generate highly sensitive biosensor electrodes.

**Figure 11:**
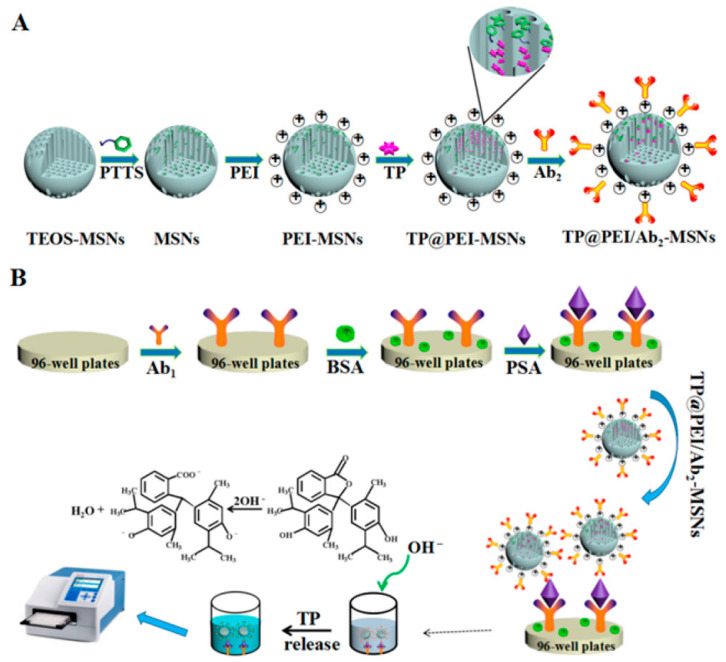
(**A**) TP@PEI/Ab2-MSNs Synthesis and Derivatization; and (**B**) Steps of the enzyme-free immunosorbent assay of PSA using TP@PEI/Ab2−MSNs for amplified colorimetric detection in a 96-well plate; and (**B**) Reaction involved in releasing the TP molecules by 0.1 M NaOH is shown at the bottom of panel (**B**) [132].

Nevertheless, Shao et al. developed an enzyme-free titer plate-based colorimetric assay utilizing functionalized mesoporous silica nanoparticles (MSNs) entrapping pH-indicator molecules [132], Figure 11.

Antibody molecules are electrostatically adsorbed onto the MSNs (the antigen used in our proof-of-concept experiment is a prostate-specific antigen), whereas colorimetric detection can be accomplished by releasing the reporter molecule (thymolphthalein, a pH-sensitive indicator) entrapped in the MSN pores into the analyte solution.

Graphene aerogels (GAs), as carriers of functional materials, enlarge the specific surface area and increase the conductivity of the material, and thus improve the electrocatalytic properties of composite materials. Based on these excellent properties, GAs can be employed on different uses for antibodies-biosensor. For instance, highly sensitive immunosensor for the cancer marker carbohydrate antigen 15–3 (CA15–3) was designed by Jia et al., it was based on the use of polymeric β-cyclodextrin (Pβ-CD)-GAs [133].

The large specific area of GAs warrants high loading with antibodies, and their excellent electrical conductivity warrants strong electrical signals. The response is linear in the 0.1 mU mL^−1^ to 100 U mL^−1^ activity range, and they obtained a lower detection limit, 0.03 mU mL^−1^ (at S/N = 3). The immunoassay was stable, selective and reproducible.

The 3D-GA was prepared via in situ chemical reduction of graphene oxide with L-ascorbic acid and then dehydration by freeze-drying by Hu et al. [134]. After the modification of the 3D-GA, they were be used for detecting various tumor markers in liquid samples via electrochemical impedance spectroscopy (EIS). The electrochemical platform achieved a broad detection range of 1.0 × 10^−8^–1.0 × 10^−5^ and 1.0 × 10^−8^–5.0 × 10^−4^ mg mL^−1^ for alpha-fetoprotein (AFP) and carcinoembryonic antigen (CEA), respectively, and a low limit of detection (LOD) of 7.9 and 6.2 pg mL^−1^ for AFP and CEA respectively.

Finally, an electrochemical sensor for detection of cancer cell based on folic acid (FA) and octadecylamine (OA)-functionalized graphene aerogel microspheres (FA-GAM-OA) has been investigated [87]. The freeze drying retains the porous structure and the thermal reduction process enhances the conductivity of the graphene-based material leading to great sensitivity. The FA-GAM-OA offers a large surface area (1723.6 m^2^ g^−1^) and high electronic conductivity (2978.2 S m^−1^). The electrochemical sensor based on FA-GAM-OA exhibits extremely good analytical performances in detection of liver cancer cells with a linear range of 5–10^5^ cell mL^−1^ giving a low detection limit of 5 cells mL^−1^ (S/N = 3), showing an exceedingly large specific surface area and great selectivity to cancer cells.

### 4.3. Aptamer-Based Biosensor

Aptamers are single-stranded DNA or RNA sequences, with high affinity to a wide range of targets, including small molecules, ions and proteins, therefore, attractive for biosensors development. They typically have a length of a hundred base pairs compromised of 20–70 randomized base pair binding region in the center and with constant primer binding regions at both ends. Aptamers have been used as bio-recognition elements due to their good chemical stability, structure feasibility, small size, high specificity, flexible design, and ease of chemical modification [136,137]. Especially, the affinities of aptamers for their targets are reported to be comparable to, or even higher than most monoclonal antibodies.

Taking advantages of aptamers and aerogels properties, some sensor systems combining both materials have been reported. Table 4 summarizes the aptamer-based biosensors, incorporating aerogels in the system.

**Figure 12:**
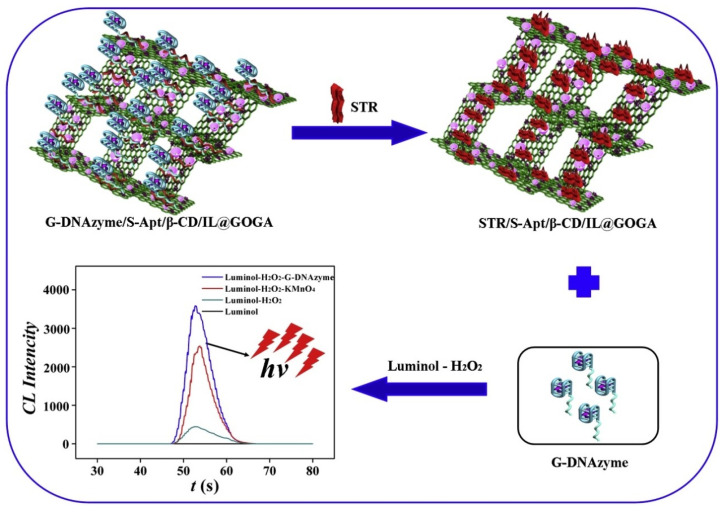
Illustration of principle diagram of CL streptomycin sensor [138].

A chemiluminescence biosensor for insulin detection was proposed by Sun et al. [96] based on aptamer and ssDNA-AuNPs functionalized nanosilica-graphene oxide aerogel. When insulin is present, it will bind to the insulin aptamer, resulting in the release of ssDNA-AuNPs, which will catalyze the chemiluminescence reaction of luminol-H_2_O_2_ and increasing the luminescence intensity. Insulin aptamer was used as a biorecognition element to improve the selectivity and ssDNA-AuNPs was used as a catalyst for luminol-H_2_O_2_ to improve the sensitivity of the developed biosensor. Under optimal conditions, an ultra-low LOD of 1.6 pM was obtained. The same authors also prepared a highly selective streptomycin chemoluminescense sensor, shown in Figure 12, an important aminoglycoside antibiotic, based on aptamer and G-quadruplex DNAzyme modified graphene composite [138]. In this case, graphene oxide aerogel was used as a skeleton material to provide higher specific surface area, and then functionalized with β-cyclodextrin rich in hydroxyl groups and ionic liquid stable ions to increase the biocompatibility and stability of the composite.

After that tetracycline aptamer and G-DNAzyme was immobilized on the surface of the obtained platform. The aptamer with specific recognition ability to streptomycin and G-quadruplex-DNAzyme as a catalyst of luminol-H_2_O_2_ were combined to obtain a chemiluminescence system with improved selectivity and sensitivity. In the presence of streptomycin, due to the binding ability between streptomycin and the aptamer, G-quadruplex-DNAzyme was released from the surface, catalyzing the chemoluminescence reaction. The sensor showed a detection limit of 9.2 × 10^−14^ mol/L.

**Figure 13:**
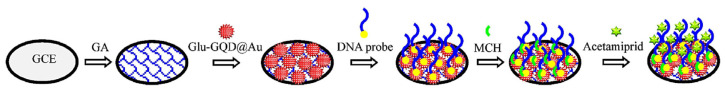
Scheme for fabrication of aptasensor for detection of acetamiprid [139].

Regarding electrochemical detection, a biosensor based on glutamic acid-functionalized graphene quantum dots with Au seeds was prepared for the determination of acetamiprid [139], Figure 13. The resulting surface was covalently connected with the acetamiprid aptamer to obtain a redox probe with catalyst activity. The aptamer can specifically bind with acetamiprid and produce sensitive and selective electrochemical response.

The electrochemical aptasensor exhibits ultrahigh sensitivity and selectivity for detection of acetamiprid. The DPV signal linearly decreases with increasing acetamiprid concentration in the range from 1.0 fM to 1 × 10^5^ fM with a detection limit of 0.37 fM.

A new electrochemical aptamer biosensor for the platelet-derived growth factor (PDGF-BB) detection, an important regulator of cell growth and di vision, has been developed [141].

**Figure 14:**
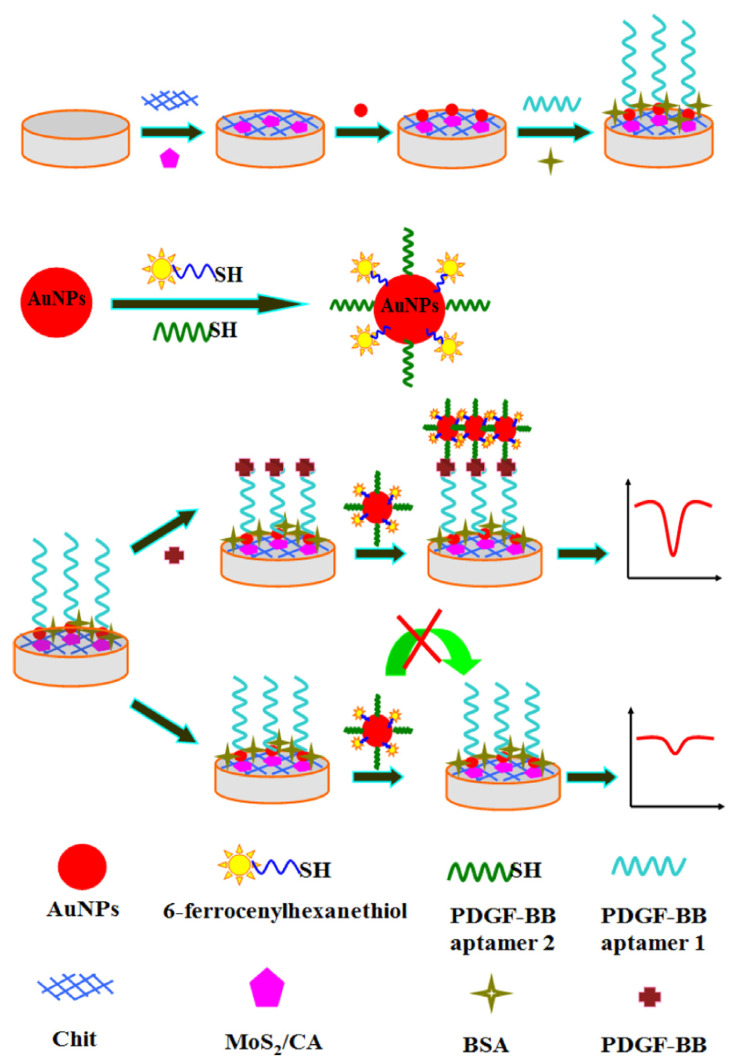
Schematic diagram of the electrochemical aptamer biosensor based on AuNPs and MoS2/CA signal amplification for the detection of PDGF-BB [141].

Carbon aerogel incorporated molybdenium nanostructures, with large surface-active sites, was placed in a GC electrode, Figure 13. This surface was then modified with aptamer-1 (thiol terminated PDGF-BB/AuNPs). At the same time, AuNPs with thiol-terminated PDGF-BB (aptamer-2) and 6-ferrocenylhexanethiol were also prepared, as shown in Figure 14.

By sandwiching the PDGF-BB between these both systems, a signal amplification of a sandwich assay was observed using DPV as detection technique. A linear response is observed in the range of 0.001–10 nM, with a LOD of 0.3 pM.

Mycotoxins are toxic contaminants produced by the secondary metabolism of fungi. Ochratoxin A, one of the highly toxic mycotoxins, secreted by Aspergillus and Penicillium has attracted increased attention since it contaminates agricultural products [142]. Based on the signal amplification strategy, an aptamer-based electrochemical biosensor was developed for the determination of Ochratoxin A by using carbon aerogels and methylene blue as signal amplification [142].

Carbon aerogel was used as a carrier to which complementary DNA was linked. By enhancing the hybridization between carbon aerogel-complementary DNA, and aptamer immobilized on the Au electrode surface, more double-stranded DNA for methylene blue intercalation was provided. The higher loading capacity of the complementary DNA, results in more absorbed methylene blue, thus increasing the signal amplification strategy. If Ochratoxin A is present, a proportional amount of methylene blue is release, originating a change of peak current, which was linearly proportional to the Ochratoxin A concentration in the range of 0.10–10 ng/mL with the actual detection limit of 1.0 × 10^−4^ ng/mL.

Carbendazim, a broad-spectrum fungicide, has been used in fresh food production to prevent pathogens and pest’s proliferation. However, this compound may cause serious environmental pollution through long-time accumulation in plants. A high level of carbendazim in food has been associated with some side effects in humans, such as endocrine disorders and cancers. This way, an aptasensor for electrochemical detection of carbendazim was developed by Jin and co-workers [140] with gold nanocrystal/multiple graphene aerogel and DNA cycle amplification. The gold nanocrystal underwent structural evolution under enantioselective direction of L-cysteine. The exposure of high-index facets improves the catalytic activity. This system was used for the construction of an aptasensor for electrochemical detection of carbendazim. The aptamer hybridizes with assistant strandDNA to form duplexDNA. Carbendazim binds with the formed duplex DNA to release assistant strand DNA, triggering one three-cascade DNA cycle.

The utilization of a DNA cycle allows one carbendazim molecule to bring many methylene blue–labeled DNA fragments to the electrode surface. This promotes significant signal amplification due to the redox reaction of methylene blue. The detection signal is further enhanced by the catalysis of gold nanocristals and graphene aerogel towards the redox of methylene blue.

A differential pulse voltammetric signal, increases linearly with the carbendazim concentration ranging from 1.0 × 10^−16^ to 1.0 × 10^−11^ M with a detection limit of 4.4 × 10^−17^ M.

### 4.4. Aspects of the Bio-Recognition Elements

Biosensors can be categorized, according to the biorecognition principle, as a catalytic and affinity or non-catalytic biosensors. In the first case, the analyte interaction with the bioreceptor results in a new product, as itis the case of enzyme-based biosensors. In the case of affinity biosensor, the analyte is irreversibly bound to the receptor and the interaction does not result in the formation of a new product. This type of sensor comprises antibodies and nucleic acids, such as aptamers [143,144].

Enzymes are the most common biorecognition elements. They are used as a biocatalyst to increase the biological reactions rate, producing various measurable products. An enzyme-based biosensor is based on the catalytic reaction and binding capabilities for the target analyte detection, and can work in two main different ways. The analyte is metabolized by the enzyme, resulting in a new product. In this case, the concentration is obtained by measuring the catalytic transformation of the analyte by the enzyme. In the second case, the enzyme is inhibited by the analyte, and the analyte concentration is related to the decrease in the enzymatic product formation [143]. These processes enable very sensitive detection, resulting in a very low LOD [145].

Antibodies are another type of biorecognition element which has been used due to the strong antigen–antibody interactions [143]. Biosensors that use antibodies as ligands in the antibody–antigen interaction are called immunosensors. Immunosensors can be classified as non-labeled, those are constructed to specifically determine the antigen–antibody complex, and labeled where a sensitively detectable label is introduced. The antigen–antibody complex is sensitively assessed through label measurement [143].

Aptamers are synthetic short single-stranded DNA or RNA sequences, that bind to target analytes, such as bacteria, proteins, toxins and hormones, with high sensitivity and specificity. The recognition of the analyte by this biosensor (aptasensor) is not based on the identification of the DNA sequence, but by shape instead [144]. They can be folded into two-dimensional and three-dimensional structures. In these structures, the targets have high-binding performance due to greater surface density and less spatial blocking [145].

Unlike antibodies that require biological systems to be generated, due to the nucleic acid character of aptamers, these bio-elements are structurally and functionally stable over a wide range of temperatures and storage conditions. Aptamers can be chemically synthesized, are stable in a wide pH range (2–12), and have certain thermal refolding capabilities [143]. Another important property of aptamers is that they can be chemically modified according to the target molecule. The use of aptasensors for the detection of bacterial pathogen provides various advantages such as easy preparation, facile modification, and good stability. However, small species like proteins are preferred over large targets, such as bacteria, as a single aptamer can produce a false negative result [144,145]. To overcome this, the use of more than one aptamer assembled in a 2D or 3D structure can be a strategy to avoid the mentioned problem.

Over the last decades, aptamers and molecularly imprinted polymers (MIPs) have emerged as promising biomimetic receptors for use in biosensors as viable alternatives to natural antibodies and enzymes. They present superior stability, comparable or even superior binding performance, lower costs and have shown extended shelf lives. The tailorability of aptamers makes them particularly useful as biomimetic elements in biosensors development. The development of MIPs for small molecules is considered the best choice, since aptamers have shown a difficulty binding capacity for small molecules. Regarding aptamers development, there is a need for modified aptamers, capable to increase the aptamer-target binding interactions. For this, simplified and cost-effective methods for aptamers modification remains a challenge [146].

Regarding MIPs, significant challenges remain in the development of larger targets, such as proteins and whole cells, which suffer from a lack of available water-soluble functional monomers, while still showing significant non-specific binding and lack of access for large templates to their respective recognition sites. Moreover, template leakage which means that some of the template is not removed after synthesis, can lead to false positive results. With the increase in the number of companies that offer MIPs and aptamers solutions, the availability and costs will continue to decrease [146].

## 5. Conclusions and Future Trends

The 2030 Agenda for Sustainable Development, adopted by all United Nations Member States in 2015, provides a shared blueprint for peace, prosperity, and opportunity for all on a healthy planet. The aerogel biosensors can be utilized for rapid and accurate disease diagnostics, facilitating the early detection and treatment of various health conditions. This technology can improve access to healthcare in remote or resource-limited areas (SDG 3: Good Health and Well-being). The development and implementation of biosensors for rapid and cost-effective disease diagnostics can contribute to early detection and improved healthcare outcomes. Biosensors can be designed to detect various diseases, including infectious diseases (e.g., malaria, HIV, COVID-19) and chronic conditions (e.g., diabetes).

Biosensors offer a versatile alternative for sensing applications in several relevant fields, such as medicine and biomedicine, toxicology and ecotoxicology, food safety monitoring, drug delivery, and disease progression control. In this review, recent progress in the development of different aerogel-based sensors for biomedical applications is summarized.

An aerogel-based biosensor is an innovative technology that combines the properties of aerogels, which are lightweight and highly porous materials, with biosensors, which are devices used to detect and analyze biological or chemical substances. The integration of aerogels into biosensor devices offers several advantages. Aerogels provide a large surface area for sensing molecules, allowing for increased sensitivity and efficiency in detecting analytes. The bio-elements provide high affinity and selectivity. The role of different bio-elements, such as enzyme-based, antibody-based and aptamer-based biosensors with aerogels and the detailed biosensing principles were explored. Although impressive achievements have been realized, there are still several important challenges in the development of biosensors. Aerogel biosensors have the potential to revolutionize healthcare and related fields by offering highly sensitive, selective, and versatile platforms for detection and monitoring applications. Their unique properties make them promising candidates for addressing various challenges in diagnostics, monitoring, and environmental sensing. Continuous investment in research and development in these areas will contribute to the evolution of biosensors, such as the development of biosensors capable of detecting multiple analytes simultaneously or integrating artificial intelligence (AI) and machine learning algorithms with biosensors to enhance data analysis and interpretation, making them increasingly reliable, versatile and applicable across diverse domains.

Due to the limited availability and high cost of production, the use of biosensors still presents some issues. From the choice of the bio-element and during the development of the biosensor, some aspects need to be taken into consideration. Enzyme structure is very sensitive to pH and temperature conditions, which makes it expensive and complicated to improve its sensitivity, stability, and adaptability. In the case of antibody-based biosensors, they can take several hours to prepare; this may be a problem in cases where rapid detection is a requirement. The development of biomimetic receptors using molecular imprinted polymers (MIPs) can be used as an alternative for biosensor development [147]. MIP production is typically based on facile synthesis, has a lower production cost, and also achieves excellent selectivity.

## Figures and Tables

**Table 1:** Recent research based on aerogels for biosensors in the biomedical field.

Aerogel Matrix	Analyte/Stimuli	Detection Limit	Lineal Range	Biomedical Application	Reference
Carbon	Body pressure	1.0 Pa	0–10 kPa	Monitoring biosignals	[80]
Carbon nanofiber	pH in wound	−40.4 mV/pH	-	Chronic wound monitoring—Smart wound dressing	[81]
Graphene oxide	Quercetin	0.065 μmol/L (3S0/S)	0.1 μmol/L–100.0 μmol/L	Drug detection and quantification	[82]
MWCNTs/Mo nanoparticles	Dopamine	1.26 nM	0.01µM–1609 µM	Diagnosis and prevention	[83]
Graphene/Au	Carcino embryonic antigen	0.15 pg/mL (S/N = 3)	0.5 pg/mL–20 ng/mL	Immunosensing	[84]
Graphene oxide/Au	Uric acid	3.7 μM (S/N = 3)	5–600 μM	Metabolite monitoring	[85]
Graphene	Glucose	0.87 mM (S/N = 3)	1 mM–18 mM	Prevention and clinical diagnosis	[86]
Graphene microspheres	Cancer cells	5 cell/mL (S/N = 3)	5–10^5^ cell/mL	Cancer detection, prevention and early treatment	[87]
Amino silica	Human interleukin-6 (IL6)	0.00001 ng/ml	-	Antigen recognition	[88]
Silica	Nucleotide acids (human gene ATP5O)	-	0.1–10 μM	Gene recognition	[89]
Peptide-Nanocellulose	Human neutrophil elastase	0.13 units/milliliter	-	Biomarker for inflammatory diseases	[90]
Protein-Nanocellulose	Copper ions	200 × 10^−9^ M	-	Point-of-care diagnostics	[91]
Poly(vinyl alcohol)	Alcohol	0.50 mM (saliva)	0–40 mM	Point-of-care diagnosis	[92]
Gold nanowire	Ethanol	0.01 M	0.01–0.5 M	Disease diagnosis	[93]
Palladium	Glucose	2 mM	2–20 mM	Prevention and clinical diagnosis	[94]
Au/Pt	Organophosphorus compounds	0.185 ng/L	0.5–1000 ng/L	Medical diagnosis	[95]
Nanosilica/Graphene oxide	Insulin	1.6 × 10^−12^ moL/L	7.5 × 10^−12^–5.0 × 10^−9^ moL/L	Medical treatments and injections	[96]
CNTs/MoSx	Avian Influenza Virus H7	0.43 ng/mL	1–25 ng/mL	Immunosensing	[97]

**Table 3:** Antibody-based biosensors in aerogel matrices.

Aerogel	System	Target	Detection Technique	Linear Range	LOD	Reference
Carbon nanotube aerogel	Ethylenediamine grafted carbon nanotube aerogels modified screen-printed electrode	Alpha-fetoprotein (AFP) and carcinoembryonic antigen (CEA)	Square Wave Voltammetry (SWV)	5.0 × 10^−12^–1.0 ng/mL	0.0010 ng mL^−1^	[129]
Cellulose nanofiber aerogel	Cellulose nanofiber (CNF) aerogel material incorporated into LFIA strips	IgG	Lateral flow immunoassays	0.72 ngmL^−1^–100 ngmL^−1^	4.6 ng mL^−1^–100 ng mL^−1^ (in human serum)	[130]
AuNPs/nano-PEDOT-graphene aerogel(GA)	Three-dimensional (3D) structural nano-PEDOT-graphene aerogel (nano-PEDOT-GA) composite	Metformin	Differential pulse voltammetry (DPV)	0.0001–50 ng mL^−1^	0.03 pg mL^−1^	[131]
Mesoporous silica	Releasing pH Indicator Molecules Entrapped in Mesoporous Silica Nanoparticles	Prostate specific antigen	Calorimetric	0.5–8000 pg mL^−1^	0.36 pg mL^−1^	[132]
Graphene aerogel	Graphene aerogel with β-cyclodextrin polymer (Pβ-CD) for immobilization of antibodies	Carbohydrate antigen	Differential pulse voltammetry (DPV) and Electrochemical Impedance Spectroscopy (EIS)	0.1 mU mL^−1^–100 U mL^−1^	0.03 mU mL^−1^	[133]
Graphene aerogel	Graphene aerogel via in situ chemical reduction of graphene oxide with L-ascorbic acid and then dehydration by freeze-drying	Alpha-fetoprotein	Electrochemical impedance spectroscopy (EIS)	1.0 × 10^−8^–1.0 × 10^−5^ mg mL^−1^	7.9 pg mL^−1^	[134]
Graphene aerogel microspheres	Folic acid (FA) and octadecylamine (OA)-functionalized graphene aerogel microspheres (FA-GAM-OA)	HepG2	Cyclic voltammetry (CV) and differential pulse voltammograms (DPV)	5–10^5^ cell mL^−1^	5 cells mL^−1^	[87]
Graphene aerogel	Immobilization of aptamers on the screen-printed electrode (SPE) surface modified by GA/AuNPs/Nafion.	Prostate specific antigen	Electrochemical impedance spectroscopy	0.05–50 ng∙mL^−1^	0.0306 ng∙mL^−1^	[135]

**Table 4:** Aptamer-based biosensors in aerogel matrices.

Aerogel	System	Target	Detection Technique	Linear Range	LOD	Reference
Graphene oxide	Aptamer and oligonucleotide-AuNPs functionalized nanosilica @ graphene oxide aerogel	Insulin	Chemiluminescence	7.5 × 10^−12^–5.0 × 10^−9^	1.6 × 10^−12^	[96]
Graphene oxide	aptamer and G-quadruplex DNAzyme modified tgraphene composite	Streptomycin	Chemiluminescence	1.4 × 10^−12^ to 2.8 × 10^−9^	9.2×10^−14^	[138]
Graphene aerogel	Glutamic acid-functionalized graphene quantum dots/Au aerogel covalently connected with aptamer	Acetamiprid	Differential pulse voltammetry (DPV)	1.0 fM–1 × 10^5^ fM	0.37 fM	[139]
Graphene aerogel	Gold nanocrystal/multiple graphene aerogel and DNA cycle amplification	Carbendazim	Differential pulse voltammetry (DPV)	1.0 × 10^−16^–1.0 × 10^−11^ M	4.4 × 10^−17^ M	[140]
Carbon aerogel	Electrochemical dual-aptamer-based sandwich biosensor using molybdenum disulfide/carbon aerogel and AuNPs	platelet-derived growth factor (PDGF-BB)	Differential pulse voltammetry (DPV)	0.001–10 nM	0.3 pM	[141]
Carbon aerogel	Carbon aerogel loaded with complementary DNA and aptamer immobilized on the Au electrode surface and methylene blue as signal amplification	Ochratoxin A	Differential pulse voltammetry (DPV)	0.10–10 ng/mL	1.0 × 10^−4^ ng/mL	[142]
